# Congenital Unilateral Deafness Affects Cerebral Organization of Reading

**DOI:** 10.3390/brainsci3020908

**Published:** 2013-06-05

**Authors:** Roberta Adorni, Mirella Manfredi, Alice Mado Proverbio

**Affiliations:** Department of Psychology, University of Milano-Bicocca, Piazza dell’Ateneo Nuovo 1, Milan 20126, Italy; E-Mails: mirella.manfredi@unimib.it (M.M.); mado.proverbio@unimib.it (A.M.P.)

**Keywords:** ERPs, LORETA (low resolution electromagnetic tomography), N170, ventral occipito-temporal (vOT) cortex, reading, deafness, hemispheric asymmetry, neuroplasticity

## Abstract

It is known that early sensory deprivation modifies brain functional structure and connectivity. The aim of the present study was to investigate the neuro-functional organization of reading in a patient with profound congenital unilateral deafness. Using event-related potentials (ERPs), we compared cortical networks supporting the processing of written words in patient RA (completely deaf in the right ear since birth) and in a group of control volunteers. We found that congenital unilateral hearing deprivation modifies neural mechanisms of word reading. Indeed, while written word processing was left-lateralized in controls, we found a strong right lateralization of the fusiform and inferior occipital gyri activation in RA. This finding goes in the same direction of recent proposals that the ventral occipito-temporal activity in word reading seem to lateralize to the same hemisphere as the one involved in spoken language processing.

## 1. Introduction

Neuroscience literature has grown rich in studies demonstrating that in most individuals, the brain areas involved in word reading are lateralized to the left cerebral hemisphere [1]. For instance, neurometabolic studies typically highlight the critical role of a ventral occipito-temporal (vOT) region around the left occipito-temporal sulcus at the junction between the inferior temporal gyrus and the fusiform gyrus in orthographic processing [2,3,4]. The involvement of the left occipito-temporal regions in orthographic processing is also well documented in the electrophysiological literature. It is a common finding that the bioelectrical activity reflecting orthographic analysis first peaks at about 200 ms, through a negative peak over the left posterior regions [5]. This negative peak, identified by most authors as the so-called N170, is the first event-related potential (ERP) component sensitive to word orthographic properties [6,7]. Indeed, it has been shown that the N170 distinguishes between orthographic (consonant strings, pseudo-words, words) and non-orthographic stimuli (shapes, symbols, pseudo-letters [6,8]) or between words and pseudo-words [9]. It has also been reported that the N170 distinguishes between low *versus* high frequency words [10], especially in the case of short words [11]. As for the source of the N170, most authors agree that it might reflect the electromagnetic activity of the left vOT region [12,13]. Recent ERP studies from our research group go in the same direction. For example, we recorded ERPs to words in standard or mirror orientation to investigate the role of visual word form while participants were engaged in a letter decision task (they had to decide whether or not the stimuli contained a target letter, [14]). We found an early effect of word orientation at about 150–200 ms, with larger N170 amplitudes to rotated compared to standard words. This component was also affected by the selective attention to letters, being greater to target than to non-target words at left lateral occipital sites, thus reflecting the first stage of orthographic processing. Low resolution electromagnetic tomography (LORETA) source reconstruction revealed a strong focus of activation for this effect in the left fusiform gyrus. In another study [15] in which participants were engaged in a similar orthographic decision task, we found a larger N2 component to high-frequency than low-frequency words and pseudo-words within the left lateral occipital areas. The solution provided by LORETA suggested greater left fusiform and right superior temporal activation for processing high frequency as compared to low frequency words. In a subsequent study [16] aimed at contrasting the role of orthographic familiarity and other psycholinguistic variables while participants were engaged in a lexical decision task (they had to decide whether the stimuli were meaningful words or non-words), we demonstrated the role of orthographic familiarity in determining the early activation of the left-occipito-temporal regions, in particular, of the left fusiform gyrus. Finally, in a recent study [17], we found even earlier traces of visual-orthographic processing than the N170 component. In this study, the attentive processing of orthographic *vs.* semantic features was compared by presenting the same set of words in two different conditions: orthographic decision *vs.* lexical decision. The results evidenced that the prioritized processing of word orthographic features (during the orthographic decision task) was able to enhance the activity of left fusiform gyrus and cerebellar structures as early as 70–90 ms from stimulus-onset, as reflected by the increased amplitude of mesial C1 and lateral-occipital P1 components. After this early visual processing, N170 seems to reflect the main processes involved in orthographic analysis.

Generally speaking, the left vOT activation in word reading seems to be the neuro-functional counterpart of what cognitive models classified as the visual lexical route of reading, which directly recognizes visual word forms by means of an initial low-level visual and orthographic analysis, followed by the access to the orthographic input lexicon (see the dual route cascaded (DRC) model [18]). Following this model, neuroscientists focused on three cerebral regions of the left hemisphere. The first is the inferior occipital cortex (IOC), which is likely to be part of the initial feature and/or letter analysis. The second area is located midway along the fusiform gyrus, anterior to the IOC. Some authors refer to it as the Visual Word Form Area or VWFA [2,19], as it appears to mediate orthographic analysis [3]. The third area is located anterior to the VWFA, along the anterior fusiform gyrus. This area seems to be sensitive to lexical and semantic manipulations, and it may correspond with the DRC's orthographic input lexicon (for a review, see [20]). In this context, some researchers underline that the left vOT region is specifically dedicated to the extraction of invariant visuo-orthographic information via a posterior-to-anterior hierarchy of local combination detectors [3]. Other authors [21,22,23] suggest that neuronal populations in vOT cortex are not tuned selectively to orthographic inputs. As the starting point of their hypothesis, they consider that at the neural level, learning involves experience-dependent synaptic plasticity, which changes connection strengths. Learning to read involves linking written symbols to higher level phonological and semantic representations needed for language understanding [24]. In other words, orthographic representations emerge from the integration of visuo-spatial features abstracted from sensory inputs (bottom-up process) with top-down predictions that are conveyed by backward connections from phonological and semantic areas to vOT. This interactive account of vOT function in reading [22] states that the left lateralization of the vOT activity is a consequence of top-down connections from the anterior language areas, which are generally lateralized to the left hemisphere. Direct evidence for the relationship between language anterior cerebral regions and vOT activity during reading comes from two studies by Cai and collaborators [25,26], who compared the laterality of reading-related vOT activity in healthy individuals with typical left *vs.* atypical right hemispheric dominance for language production (right atypical language lateralization can be observed in 25%–30% of strong left-handed individuals, see [1]). By analyzing ERPs of native French readers with typical and atypical language production lateralization, Cai and collaborators [25] showed that reading-related vOT activity seems to lateralize to the same hemisphere as the one involved in spoken language production. In a subsequent study [26], they confirmed this finding using fMRI. In a recent fMRI study [27], Van der Haegen and collaborators investigated language laterality in a large sample of healthy left-handers. Participants were selected as left, bilateral or right dominant for speech production on the basis of the inferior frontal gyrus activity during a silent word generation task. Afterwards, they were asked to perform a lexical decision task, in order to test the laterality of the vOT activity. The results suggested that the lateralization of the vOT activity during the lexical decision task and the lateralization of the inferior frontal gyrus activity during the silent word generation task correlated significantly. In other words, while reading, the majority of participants showed enhanced activation of the cerebral hemisphere that was identified as dominant for word production.

In the light of this evidence, in this study, we addressed the issue of establishing if the development of the visual language cerebral regions might be influenced by the auditory canal available for linguistic listening. For this aim, we investigated the neural regions recruited during reading in a patient affected by congenital unilateral deafness with atypical language lateralization and compare them to those recruited in a group of normal hearing volunteers.

Patient RA is a woman affected by a profound unilateral congenital deafness, caused by unilateral microtia. It is a common finding that patients like RA, with unilateral microtia and with normal hearing on the contralateral side, develop normal speech [28]. It is generally accepted that the early sensory deprivation modifies brain functional structure and connectivity [29]. Auditory hemispheric patterns have been shown to change or reorganize with sound deprivation (for a review, see [30]). For instance, in a recent fMRI study [31], Firszt and collaborators found a strong left cortical asymmetry in response to speech stimuli in a group of normal-hearing volunteers and a notable decrease in asymmetry in a group of patients affected by acquired unilateral hearing loss. For this group, the asymmetry reduction was a result of both a decrease in the left hemisphere and an increase in the right hemisphere activity. Many researchers have argued for the ‘‘equipotentiality’’ of the two hemispheres, and there is evidence that the right hemisphere can carry out language processes normally attributed to the left hemisphere [32]. For example, a certain percentage, even though quite small (5% of right-handed and 30% of left-handed people), of healthy individuals are right hemisphere dominant for language [27]. Also, unilateral left hemisphere damage in childhood can change the hemispheric dominance for language [33]. This issue is discussed in depth by Locke [32]. According to Locke’s theory, it can be the case that cognitive under-stimulation or lexical deprivation during the language learning period, as well as other mechanisms that cause an inactivation of the left language cerebral regions, can induce selective growth of right hemisphere homologues areas and, thus, reduce asymmetry across the two hemispheres.

Taking into account this evidence and considering that it is well-known that the pathway from each ear to the contralateral cortical hemisphere comprises more nerve fibers than the pathway from each ear to the ipsilateral hemisphere [30], we approached the investigation of RA’s case. Because of her congenital auditory sensory deprivation, RA presumably has a right lateralization of word phonological properties processing. On the other hand, visual word processing is supported by a normal visual system, so that it was not possible to advance a hypothesis about the organization of vOT activity during reading. Therefore, an atypical organization of vOT activity in reading, considering that it was not forced by her sensory deprivation (the vOT activity reflects the visual processing of stimuli, and RA has a hearing, not a visual deficit), may provide support to the hypothesis that word reading lateralizes to the same hemisphere as the one involved in spoken language processing.

In this study, we used the ERP technique, and we employed an original paradigm in which participants were asked to visually detect a target letter (orthographic decision task), thus focusing on the orthographic processing of words. We expected that orthographic analysis enhanced the activity of the visual cortex at an early processing stage (presumably in the latency range of the N170 component). By means of the standardized LORETA (swLORETA) inverse solution, we aimed at identifying brain regions that were activated in response to visually presented words, in order to investigate whether RA may have a different hemispheric lateralization of orthographic processing with respect to normal hearing volunteers, thus providing support (or not) to the hypothesis that word reading lateralizes to the same hemisphere as the one involved in spoken language processing.

## 2. Results and Discussion

### 2.1. Behavioral Results

#### 2.1.1. Control Participants

The analysis performed on mean response times (RTs) of the control group evidenced no effect of the response hand. Mean RTs were 550 ms (SD = 66) for the right hand and 557 ms (SD = 69) for the left hand. The percentage of errors of the control group was very low (mean false alarms = 0.7%; mean omissions = 0.7%), and no effect of the response hand was found.

#### 2.1.2. Patient RA

Mean RTs of RA were 518 ms for the right hand and 548 ms for the left hand. She performed no errors.

### 2.2. Electrophysiological Results: Occipito-Temporal N170 Component

#### 2.2.1. Control Participants

**Figure 1:**
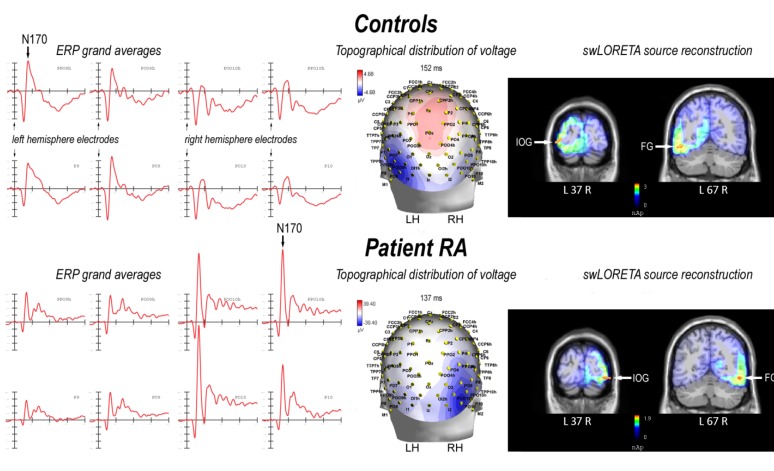
On the left: grand-average event-related potential (ERP) waveforms recorded at left (PPO9h, POO9h, P9, PO9) and right (PPO10h, POO10h, P10, PO10) occipito-temporal electrode sites in response to words in controls (top) and RA (bottom). In the middle: back view of the topographical distribution of voltage recorded in response to words in controls (top) and RA (bottom) in correspondence to the N170 peak latency (LH = left hemisphere, RH = right hemisphere). On the right: coronal views of N170 active sources for the processing of words in controls (top) and RA (bottom), according to the standardized low resolution electromagnetic tomography (swLORETA) source reconstruction. The figure shows the dipoles corresponding to the activation of the inferior occipital gyrus (IOG) and the fusiform gyrus (FG).

The N170 component reached its maximum amplitude over the occipito-temporal regions of the left hemisphere, as visible in the ERP waveforms and topographic maps of Figure 1. The analysis performed on the peak amplitude of the N170 confirmed the left lateralization of the cerebral activation at the scalp surface (left hemisphere (LH) = −6.12; right hemisphere (RH) = −3.17 µV), as evidenced by a significant effect of hemisphere (*F*(1,14) = 17.03; *p* < 0.005). N170 was also affected by the presence of the target letter (*F*(1,14) = 7.52; *p* < 0.05), showing larger amplitudes in response to target compared to non-target words (T = −5.00; NT = −4.29 µV). The analysis performed on the peak latency of the N170 evidences no effect of the factors considered in this study. The mean peak latency of the N170 was 168 ms over the left hemisphere and 169 ms over the right hemisphere.

SwLORETA source reconstruction was performed in the peak latency range of the occipito-temporal N170 (between 140 and 180 ms). The solution showed a strong activation of the neural circuit within the extra-striate visual areas of the ventral pathway, namely the inferior occipital gyrus (IOC, Brodmann Area (BA) 18) and the fusiform gyrus (FG, BA 19). This activation was left-sided (see Table 1 for a list of electromagnetic dipoles and Figure 1).

**Table 1:** Talairach coordinates corresponding to the intracortical generators explaining the surface voltage recorded during the 140–180 ms time window in response to words in control participants. Power RMS = 51.7 µV. O, occipital; T, temporal; F, frontal.

Magnitude (E-10)	T- *x* [mm]	T- *y* [mm]	T- *z* [mm]	Hemisphere	Lobe	Gyrus	BA
28.0	−38.5	−87.3	−4.9	L	O	Inferior Occipital Gyrus	18
27.7	−48.5	−66.1	−10.9	L	T	Fusiform Gyrus	19
11.8	21.2	−16.1	−22.2	R	Limbic	Parahippocampal Gyrus	28
6.51	−8.5	57.3	−9	L	F	Superior Frontal Gyrus	10
6.26	1.5	38.2	−17.9	R	F	Medial Frontal Gyrus	11
5.12	11.3	57.3	−9	R	F	Superior Frontal Gyrus	10

#### 2.2.2. Patient RA

The N170 component reached its maximum amplitude over the occipito-temporal regions of the right hemisphere (LH = −10.94; RH = −30.27 µV), as visible in the ERP waveforms and topographic maps of Figure 1. Similarly to the control group, the N170 was larger in response to target compared to non-target words (T = −21.13; NT = −20.07 µV). The mean peak latency of the N170 was 136 ms over the left hemisphere and 137 ms over the right hemisphere.

swLORETA source reconstruction was performed in the peak latency range of the occipito-temporal N170 (between 120 and 150 ms). Similarly to the control group, the solution showed a strong activation of the neural circuit within the extra-striate visual areas of the ventral pathway, namely the inferior occipital gyrus (IOC, BA 18) and the fusiform gyrus (FG, BA 19). As opposed to the control group, in RA, this activation was strongly right-sided (see Table 2 and Figure 1).

**Table 2:** Talairach coordinates corresponding to the intracortical generators explaining the surface voltage recorded during the 120–150 ms time window in response to words in RA. Power RMS = 282.8 µV. O, occipital; T, temporal; F, frontal.

Magnitude (E-10)	T- *x* [mm]	T- *y* [mm]	T- *z* [mm]	Hemisphere	Lobe	Gyrus	BA
187	40.9	−86.4	−12.4	R	O	Inferior Occipital Gyrus	18
180	50.8	−66.1	−10.9	R	T	Fusiform Gyrus	19
64.6	−18.5	−24.5	−15.5	L	Limbic	Parahippocampal Gyrus	35
64.6	−28.5	−15.3	−29.6	L	Limbic	Uncus	20
35.0	−8.5	57.3	−9	L	F	Superior Frontal Gyrus	10
28.7	1.5	38.2	−17.9	R	F	Medial Frontal Gyrus	11
25.5	11.3	57.3	−9	R	F	Superior Frontal Gyrus	10
6.78	1.5	29.5	58.7	R	F	Superior Frontal Gyrus	6

### 2.3. N170 Laterality Index

To evaluate the difference in the lateralization of the N170 component between patient RA and controls, for all the participants, we calculated the normalized difference (laterality index or LI = (LH − RH)/(LH + RH)) between the amplitude of the N170 in response to word stimuli at the left temporal occipital electrodes (PPO9h, POO9h, P9, PO9) and the right temporal occipital electrodes (PPO10h, POO10h, P10, PO10). Control participants had a mean laterality index of 0.33 (the N170 reached its maximum amplitude over the left hemisphere); RA had a mean laterality index of −0.47 (the N170 reached its maximum amplitude over the right hemisphere). A *t*-test confirmed that RA and control participants differed in terms of LI (*t*(14) = 3.33; *p* < 0.005).

### 2.4. Discussion

Both control volunteers and patient RA had a highly accurate performance on the orthographic task. Response times were marginally faster when participants (both controls and RA) responded with their right hand, but no statistically significant effect was found. Behavioral responses of patient RA were similar to those of control volunteers, supporting the evidence that she had no reading difficulties whatsoever.

The ERP results evidenced an early negative peak in response to word stimuli over the occipito-temporal electrode sites, identifiable as an N170 component. The amplitude of the N170 was affected by the presence of the target letter, as larger responses to target than to non-target words were observed in both patient RA and the control group. Generally speaking, the early negativity recorded at posterior sites, also known as selection negativity [34], mirrors the neural activity of the visual areas known to code a given visual feature as a function of attention allocation to that feature. This effect has been described for many visual features [35,36], and it has been recently observed during written word processing. For instance, in one of the studies mentioned in the introduction [14], we found that selective attention to letters modulated the amplitude of the N170 component over the left occipito-temporal cortex. The finding of a modulation of the N170 in correspondence of an orthographic selection goes in the same direction as the electrophysiological studies, suggesting that the negativity recorded over the occipito-temporal sites may be indicative of the processing of the word orthographic properties [6,7]. As reported in the introduction, this negativity may trace the activation of a lexical pathway that runs along the inferior surface of the temporal lobe [20].

Both the peak amplitude and the topographical distribution of the N170 evidenced that this component was left-lateralized in controls, while it was strongly right-lateralized in patient RA. The results of swLORETA source reconstruction suggested that the N170 component was in all probability associated with an activation of the extra-striate visual areas of the ventral pathway, particularly of the inferior occipital gyrus (IOG, BA 18) and the fusiform gyrus (FG, BA 19). As reported in the introduction, the involvement of these two cerebral regions in orthographic and lexical processing is well documented in neuroimaging and electrophysiological literature on normal hearing right-handed readers [4,20]. In all the studies cited in this paper, in all our previous studies, as in the control group of the present study, this activation was left-lateralized. Intriguingly, the cerebral pattern of RA showed enhanced activation of the same cerebral regions (IOG and FG) of the group of normal hearing participants, but with a right instead of a left lateralization. Considering that RA was completely deaf in the right ear since birth, we can reasonably suppose that she had a right lateralization of the phonological properties of the words. Therefore, our results seem to provide support to the hypothesis that word reading lateralizes to the same hemisphere as the one involved in spoken language processing [25,26]. As already mentioned in the introduction, this hypothesis states that the left vOT region is one component of the neural system of reading, whose activation is not, however, restricted to visual word processing [23]. The precise low-level function of this posterior processing region may depend on the modulation by top-down projections from higher order association cortices. This could allow cross-modal integration of non-visual properties, such as phonology, with the visual characteristics of the stimulus [24]. According to Price and Devlin [22], the left bias of activity in the vOT is not just due to some specialization of this region for language processing, but is, at least in part, a consequence of top-down connections from the anterior language areas related to phonology and semantics, which are generally lateralized to the left hemisphere. Our results about the right lateralization of vOT activation in response to written word stimuli in patient RA seem to support this view. As already underlined, in all likelihood, RA has a right hemispheric lateralization of speech processing, considering that she is completely deaf in the right ear since birth and she is strongly left-lateralized as to earedness on the Edinburgh Inventory Questionnaire. It would be of great interest to investigate what spoken language processes are specifically lateralized to the right hemisphere in patients like RA. Previous studies focused on speech production processes and performed on a large sample of individuals (up to 250 healthy left-handed) found a little percentage of individuals with clear RH dominance for speech production [27,37]. In light of this evidence, it may be supposed that RA, as a typical right handed person, has a left hemisphere dominance of the cerebral regions involved in speech production. It cannot be excluded that, since she is affected by congenital unilateral deafness, RA may have right lateralization for speech perception processes. This would suggest that the vOT activity may co-lateralize with speech perception rather than speech production, and it might account for cases of crossed laterality between the cerebral regions involved in speech production (in particular, BA 44) and vOT activity (see [27,37]). Interestingly, a very recent study [38] aimed at investigating how atypical speech dominance is related to the structural asymmetries of the cerebral cortex, found that the main anatomical differences in grey matter between left dominant and right dominant individuals are situated in the superior temporal gyrus and vOT cortex, two regions involved in language perception in the spoken and written modalities, respectively. Further investigation will hopefully clarify this issue. Interestingly, ERP studies using the auditory mismatch negativity (MMN) paradigm to investigate the predisposition to dyslexia in at-risk infants (infants with at least one dyslexic relative) have shown a bilateral MMN to deviant phonemes in at-risk infants that did not develop dyslexia and left activation in infants that were not at-risk [39]. Moreover, a recent ERP study by Hasko and colleagues [40] that engaged a group of children affected by developmental dyslexia and a group of control children, showed that when the integration between orthographic and phonological representations was required, N300 amplitude was stronger over the left fronto-temporal hemisphere in control children. Conversely, the children with developmental dyslexia had attenuated amplitudes in the left hemisphere and enhanced amplitudes in the right hemisphere. The group differences were located in the right temporo-parietal areas, including the superior temporal gyrus (STG, BA 22), the supramarginal gyrus (SMG, BA 40), the middle temporal gyrus (MTG, BA 22) and the inferior parietal lobule (IPL, BA 40). It is generally accepted that phonological processing involves the superior temporal gyrus, orthographic processing involves the vOT cortex (as repeatedly mentioned in the present paper) and semantic representation processing involves the middle temporal gyrus [4]. The interaction among these representations are mediated by posterior heteromodal regions, including the supramarginal and angular gyrus [41]. The results of the study by Hasko and colleagues demonstrated that dyslexic children failed to recruit left temporo-parietal regions when performing tasks that require the phonological access to orthographic stimuli. On the contrary, the activation of the left vOT cortex in the latency of the N170 component was left-sided in both dyslexic and control children. In light of this evidence and considering that our results illustrate how the right vOT cortex may develop expertise normally attributed to the left hemisphere, it might be supposed, as a conjecture, that the development of the right vOT cortex for word reading might be quite helpful for reading learning in children that are predisposed to process phonemes with the right cerebral regions (see also [32,42]).

Overall, our results illustrate how the right hemisphere may develop expertise/processing skills normally attributed to the left hemisphere. They evidence the potential possibility of an alternative and efficient neuro-cognitive network, which may support orthographic processing in a deaf reader, and they enrich the neuroscience literature suggesting how early sensory deprivation can modify brain functional structure and connectivity. It is important to underline that in this study, we have described the pattern of activation of a single case, which may not necessarily generalize to all other good readers who are deaf, because of inter-individual variability. Further investigation will hopefully shed some light on this matter.

## 3. Experimental Section

### 3.1. Participants

#### 3.1.1. Case Report: Patient RA

RA is a 31 year-old Italian monolingual female affected by a profound unilateral (right ear) congenital deafness, caused by unilateral microtia. She has left normal hearing and right hearing loss (80 dB mean loss over seven octaves, spanning 125–8000 Hz). RA was a graduate-professional at the moment of electroencephalogram (EEG) recording. She had no history of neurological or psychiatric disorders. She had no history of intelligence deficits and no history of difficulty in learning to speak or to read. She had corrected-to-normal vision. The lateral preference of eyes, ears, hand and feet was assessed using the Italian version [43] of the Edinburgh Inventory Questionnaire [44]. No case of left-handedness was reported for any of her relatives. On the basis of the lateral preference questionnaire, the patient had a laterality index of 0.71 (scale −1/+1). She was strongly right-lateralized as to handedness, footedness and eyedness, while she was strongly left-lateralized as to earedness. Eye dominance was also assessed by means of two independent practical tests. One test is the so-called ‘tube’ test, in which a person looks in free vision at the experimenter through a paper tube held with both extended arms, and only the dominant eye will be visible to the experimenter. The second test involves aligning a rod (e.g., a pen) to a margin in monocular vision (e.g., the window border), while closing, in alternation, the left and right eyes and then having the subject judge under which condition the monocular image is most similar to the binocular image; the dominant eye will contribute the most to binocular vision. RA had right eye dominance at both tests. Considering that the study was focused on written word stimuli, a reading comprehension test (advanced MT Reading test, [45]) was administered to RA to ascertain that she had no reading difficulties. This test requires the participant to read two passages aloud (time and accuracy are valuated) and to respond to a total of 20 multiple-choice questions. RA’s accuracy and speed in both reading and comprehending were within the medium-high range. Phonology and comprehension of the morphosyntactic relations were assessed by means of a phonemic fluency test and a short version of the Token Test, respectively [46]; in both tests, RA’s responses were fully within the normal range.

#### 3.1.2. Control Participants

To compare RA’s ERPs with those from a control sample, ERP data from 15 normal hearing volunteers (7 males, 8 females, mean age of 26 years, SD = 10) were used. They all were right-handed, and they had a mean laterality index of 0.81 (SD = 0.18) at the laterality questionnaire. All participants were native Italian speakers and had normal or corrected-to-normal vision. They all were free of neurological or psychiatric disorders. All participants were given an interview to ensure that they did not have a history of intelligence, reading or oral-language deficits. Control participants were matched to RA for cultural status and education level (college).

The experiment was conducted with the understanding and written consent of each participant according to the Declaration of Helsinki (BMJ 1991; 302: 1194) and in compliance with the APA ethical standards for the treatment of human volunteers (1992, American Psychological Association).

### 3.2. Stimuli and Procedure

A total of 300 concrete, highly imageable Italian nouns served as stimuli. The stimuli were presented one at a time in the center of a PC monitor. They were typed in Arial Narrow capital letters and were written in white on a gray background. The words ranged from 2.5 to 9 cm in length. They were 1 cm high and subtended visual angles of 0°30′11′′ on the vertical axis and between 1°15′27′′ and 4°31′37′′ on the horizontal axis. Six blocks of trials were created. Each block lasted approximately 3 min and was preceded by 3 warning signals (“READY”, “STEADY”, “GO”) that were presented for 800 ms. Each stimulus remained on the screen for 1600 ms and was followed by a 1000–1200 ms random interstimulus interval (ISI). The participants were seated in an acoustically and electrically shielded box at a distance of 114 cm from the screen. They were instructed to fixate on a cross in the center of the screen and to minimize any eye or body movement during the recording period. The task consisted of determining, as quickly and accurately as possible, whether or not the stimulus contained a target letter suggested by the experimenter (orthographic decision task). Half the stimuli were targets in that they contained a given target letter (B, G, L, M or S) defined at the beginning of each run. The position of the target letter within the string was evenly-distributed between beginning, middle or final part. Target and non-target nouns were balanced in terms of length, varying between 3 and 10 letters (target = 7; SD = 1.6; non-target = 7; SD = 1.7). They were also balanced in terms of orthographic neighborhood density (target = 2; SD = 3.4; non-target = 2; SD = 2.9) and lexical frequency (target = 114; SD = 200; non-target = 93; SD = 121). A *t*-test was conducted for each of these parameters, and they were not significantly different at the *p* = 0.05 level. Orthographic neighborhood density was taken from a written corpus of Italian words (EPOS 2, [47]). Word frequency was taken from a comprehensive online database of Italian words (ColFIS, [48]). Participants responded by pressing a button with the index finger of one hand. Participants alternated between hands during the recording session. The order of hand use and the order in which the blocks were presented were counterbalanced across participants. Before the experimental session, the participants were given written and oral instructions about the task and were presented with two blocks of training trials similar to the experimental trials.

### 3.3. EEG Recording and Analysis

The electroencephalogram (EEG) was continuously recorded from 128 scalp sites at a rate of 512 Hz using tin electrodes mounted in an elastic cap (Electro-Cap) and arranged according to the international 10-5 system [49]. To monitor blinks and vertical eye movements, two electrodes were placed below and above the right eye (vEOG channel). Horizontal movements were monitored by two electrodes placed at the outer canthi of the eyes (hEOG channel). Averaged ear references were used. The EEG was recorded using EEProbe recording software (ANT Software, Enschede, The Netherlands) and was amplified using an ANT amplifier with a half-amplitude band pass of 0.016–100 Hz. Electrode impedance was kept below 5 kΩ. The EEG was analyzed using EEProbe software (ANT Software, Enschede, The Netherlands). Computerized artifact rejection was performed before averaging to discard epochs in which eye movements, blinks or excessive muscle potentials occurred. The artifact rejection criterion was a peak-to-valley amplitude exceeding ±50 μV. The baseline was corrected beginning 100 ms before the onset of the stimulus to the onset of the stimulus. ERPs were averaged offline from 100 ms before to 1000 ms after the presentation of the stimulus. EEG epochs were synchronized with stimulus onset, and ERP trials associated with an incorrect behavioral response were excluded from further analysis. After the offline averaging, ERPs were treated with a 40 Hz low-pass filter. Topographical voltage maps of ERPs were made by plotting color-coded isopotentials obtained by interpolating voltage values between scalp electrodes at specific latencies. low resolution electromagnetic tomography (LORETA) [50] was performed on ERP difference waves at specific latencies using ASA4 software (ANT Software, Enschede, The Netherlands). LORETA is a discrete linear solution to the inverse EEG problem that corresponds to the 3D distribution of neuronal electric activity by analyzing and mapping maximum similarity (*i.e.*, maximum synchronization) between neighboring neuronal populations (represented by adjacent voxels) in terms of orientation and strength. In this study, an improved version of standardized LORETA (swLORETA) was used that incorporates a singular value decomposition-based lead field weighting [51]. The source space properties included grid spacing of 5 mm and an estimated signal-to-noise ratio (SNR) of 3.

For each participant, response times (RTs) exceeding the mean ± 2 standard deviation (SD) were excluded. For the group of control participants, mean reaction times, arcsin-transformed percentages of errors and peak analysis of the N170 component were subjected to repeated-measure ANOVAs. Factors included “presence of the target letter” (target, non-target), “electrode” (PPO9h/PPO10h, POO9h/POO10h, PO9/PO10, P9/P10) and “hemisphere” (left, right) for electrophysiological data. For behavioral data, the factor was “response hand” (left, right). For RA, we followed a descriptive statistical approach and report her single data.

## 4. Conclusions

In summary, we reported a single case of a unilateral deaf patient, who, on the one hand, showed a normal level of reading skills and, on the other hand, showed a distinctive pattern of cerebral activation in orthographic performance with respect to a normal hearing group. More precisely, the cerebral pattern of this participant showed enhanced activation of the same cerebral regions of a group of normal hearing participants, but with a right instead of a left lateralization. Our results have two significant implications. Firstly, they seem to support the hypothesis that the lateralization of the vOT activity might be, at least in part, a consequence of top-down connections from the anterior language areas. Secondly, our results evidence the potential possibility of an alternative and efficient neuro-cognitive network, which may support orthographic processing in a deaf reader. We crucially demonstrate that the right hemisphere may develop expertise/processing skills normally attributed to the left hemisphere. In the case of patient RA, this atypical organization was not forced, as written word processing was supported by a normal visual system.
